# Hydroxytyrosol and olive leaf extract exert cardioprotective effects by inhibiting GRP78 and CHOP expression

**DOI:** 10.7555/JBR.32.20170111

**Published:** 2018-03-12

**Authors:** Li-xing Wu, Yu-yu Xu, Zhi-jian Yang, Qing Feng

**Affiliations:** 1. Department of Nutrition and Food Safety, School of Public Health, Nanjing Medical University, Nanjing, Jiangsu 211166, China; 2. Department of Cardiology, Jiangsu Province Hospital, the First Affiliated Hospital of Nanjing Medical University, Nanjing, Jiangsu 210000, China.

**Keywords:** myocardial infarction, olive leaf extract, hydroxytyrosol, endoplasmic reticulum stress

## Abstract

Myocardial infarction triggers massive biochemical changes, even cardiac cell death. Endoplasmic reticulum stress is involved in the pathology of myocardial infarction-mediated apoptosis. In the present study, myocardial cell line H9c2 cells were treated with cobalt chloride (CoCl_2_) to induce hypoxia. Isoproterenol was used for two successive days to induce myocardial infarction in SD rats. The cardioprotective effect of olive leaf extract (OLE) and its main constituent hydroxytyrosol and the underlying mechanisms were evaluated. The results showed that hydroxytyrosol markedly protected H9c2 cells against CoCl_2_-induced apoptosis. Hydroxytyrosol could reduce the mRNA and protein expression of GRP78 and CHOP induced by CoCl_2_ *in vitro*. *In vivo*, the decreased ejection fraction and fractional shortening, increased heart weight/body ratio, the formation of infarction, disordered cardiac muscle fibers and infiltration of inflammatory cells induced by isoproterenol could be significantly ameliorated by pretreatment with OLE for a month. Similarly, OLE could also reverse the increase of GRP78 and CHOP expression induced by isoproterenol. Therefore, OLE and hydroxytyrosol exert a cardioprotective effect through endoplasmic reticulum stress, which could be a new target for the prevention and treatment of cardiovascular diseases.

## Introduction

Myocardial infarction (MI) leads to heart failure and subsequent death^[1]^. It is still a main cause of death in the world despite advances in treatments^[2–3]^. Studies have shown that endoplasmic reticulum (ER) stress in cardiomyocytes can generate and promote cell apoptosis^[4]^. The C/EBP homologous protein (CHOP) pathway is the major signal pathway through which ER stress induces apoptosis. GRP78, one kind of heat shock protein 70, is the main protein in the CHOP pathway^[5]^. The expression of heat shock proteins is an essential way to antagonize this stress^[6]^. Studies have shown that GRP78 responds to hypoxia and ischemia effectively^[7]^. CHOP and GRP78 have been widely used as specific markers for ER stress^[8]^.

Olive leaf extract (OLE) contains abundant polyphenols including oleuropein, hydroxytyrosol and other flavonoids that have been investigated to fight against cardiovascular diseases^[9–10]^. Besides, Mediterranean diet with plentiful olive substances has been proven associated with a low incidence of cardiovascular diseases, cancer and stroke^[11–13]^. Hydroxytyrosol, one major component in OLE polyphenols, has been demonstrated to protect cardiomyocytes H9c2 against 4-hydroxynonenal induced cytotoxicity^[14]^. However, the mechanisms of hydroxytyrosol against hypoxia and MI are obscure.

MI is an imbalance between coronary blood supply and myocardial blood demand. In this study, cobalt chloride (CoCl_2_) was used as a hypoxia-inducing agent to mimic MI. Isoproterenol is a synthetic catecholamine and β-adrenergic agonist to induce MI. At a high concentration, isoprenaline produces severe stress, leading to infarct or infarct-like lesion of the myocardium^[15–16]^. In this study, isoprenaline was used to induce MI by subcutaneous injection for two successive days *in vivo*. We investigated the protective effect of OLE on MI induced by isoprenaline *in vivo*, and the protective effect of hydroxytyrosol on cardiomyocyte H9c2 induced by CoCl_2_ *in vitro* through the ER stress pathway*.*

## Materials and methods

### Reagents 

CoCl_2_·6H_2_O was purchased from Sigma Chemical Co (USA). Isoproterenol Yi Feixue Bio, Nanjing, China. Hydroxytyrosol (98%) was purchased from Aladdin Biological Reagent Company (Shanghai, China). OLE (25%) was provided by Sinolife United Biotech Company. H9c2 cells were obtained from the Cell Bank of Shanghai Institute of Biochemistry and Cell Biology. Dulbecco's Modified Eagle's Medium (DMEM) and FBS were purchased from ScienCell Research Laboratories (CA, USA). Primary antibodies against GRP78, CHOP and β-actin were purchased from Cell Signaling Technology (Danvers, MA, USA). Goat anti-rabbit IgG and goat anti-mouse IgG antibodies were purchased from ZSGB-BIO (Beijing, China). Hochest 33258 was obtained from the Beyotime Institute of Biotechnology (Shanghai, China). Primers used for quantitative RT-PCR were provided by Invitrogen (Carlsbad, Calif, USA). GRP78:(Forward-5'-TCAGCCCACCGTAACAATCAAGG-3'; Reverse-5'-CTTCCTCAGCAAACTTCTCGGCG-3'). CHOP: (Forward-5'-GCACCTCCCAAAGCCCTCGC-3'; Reverse-5'-CCGTTTCCTAGTTCTTCCTT-3')

### Cell culture and treatment

H9c2 cells were cultured in high-glucose DMEM supplemented with 10% FBS containing 5% CO_2_ at 37 °C. In order to induce cell hypoxia, 1×10^4^ cells/well were seeded in 96-well plates and cultured overnight. The cells were treated with CoCl_2 _at different concentrations (0, 50, 100, 200, 400, 600, 800 and 1,000 μmol/L) for 24 hours. Then, cell viability was measured by MTT assay. For the protective effects of hydroxytyrosol, hydroxytyrosol (0, 1, 5, 10, 20, or 40 μmol/L) was used for pretreating H9c2 cells for 24 hours before CoCl_2_ (400 μmol/L) inducement.

Then, 5 × 10^5^ H9c2 cells/well were seeded in a six-well plate with a glass coverslip in each well. The cells were treated with CoCl_2_ (400 μmol/L), hydroxytyrosol (40 μmol/L) and CoCl_2_ (400 μmol/L) plus hydroxytyrosol (40 μmol/L) for 24 hours. Cells with fragmented and condensed nuclei were determined using Hoechst 33258 staining (Beyotime) and annexin-V FITC/PI kit (KeyGEN, Nanjing, China) by flow cytometry according to the introductions.

### Animals and experimental protocols

All animal experiments were conducted in accordance with the Institutional Animal Care and Use Committee of the Jiangsu Province Institute of Traditional Chinese Medicine and written up following the ARRIVE guidelines. Male SD rats of (200±20) g were supplied by Shanghai Silaike Laboratory Animal Ltd and allowed to adapt to the laboratory conditions for one week before experiment. Rats were divided randomly into 4 groups (*n*=6). The normal control group was pretreated with physiological saline by oral gavage one month before subcutaneous injection of physiological saline for two days. The isoproterenol group was pretreated with ddH_2_O by oral gavage one month before subcutaneous injection of isoproterenol [85 mg/(kg·day)] for two days. The isoproterenol plus OLE group was pretreated with OLE [200 mg/(kg·day)] by oral gavage one month before subcutaneous injection of isoproterenol for two days, and the OLE [200 mg/(kg·day)] group was treated with OLE by oral gavage for one month before subcutaneous injection of physiological saline for two days.

### Western blotting

Cells were seeded in a six-well plate (5 × 10^5^ H9c2 cells/well) cultured in high-glucose DMEM supplemented with 10% FBS and then treated with CoCl_2_ or HT for indicated doses and time. Proteins were isolated by lysis buffer (Beyotime).

After the animals were sacrificed, the heart tissues were disrupted by homogenization on ice with lysis buffer. After centrifugation, protein extracts were collected.

Protein lysates were separated on 10% SDS-PAGE, transferred onto the PVDF membranes, and blocked with 5% non-fat milk for one hour, and then incubated with primary antibodies overnight at 4 °C and secondary antibodies for one hour at room temperature. Membranes were again washed with TBST and immunoreactive proteins were visualized using ECL Western blotting detection reagents (Cell Signaling Technology) were used to detect immunoreactive proteins^[17]^.

### Quantitative RT-PCR

Total RNA was extracted from H9c2 cells or heart tissues using RNAiso Plus (TaKaRaBio Technology, Dalian, China). Then, RNA was converted to cDNA using Prime Script TM RT Master Mix (TaKaRa). Real-time qPCR analysis for mRNA expression was performed using SYBR Green qPCR Master Mix (Yi FeiXue Bio, Nanjing, China) and ABI 7900. mRNA expression was normalized against GAPDH expression^[18]^.

### Echocardiography

After anesthetizing with 10% chloral hydrate, the rats were placed in decubitus supine position on the heat pad. The transthoracic echocardiography was carried out using GE ViVid-q ultrasound systems with 3-MHz linear transducer and 2-cm depth two-dimensional imaging (GE Systems, Hayozma, Tirat Carmel, Israel) when prewarmed echo transmission gel was applied to the hairless chest after injection of ISO for two days.

### Statistical analysis

Statistical analysis was performed using SPSS 18.0 and GraphPad Prism v5.0 (Graphpad Software Inc) software. Data were presented as mean±SD. Unpaired Student's *t *tests was used to compare the means of two groups, and one-way analysis of variance (ANOVA) was used to compare the means of at least three groups. *P*<0.05 was considered to be statistically significant.

## Results

### Protective role of hydroxytyrosol against CoCl_2_ treatment

**Fig.1:**
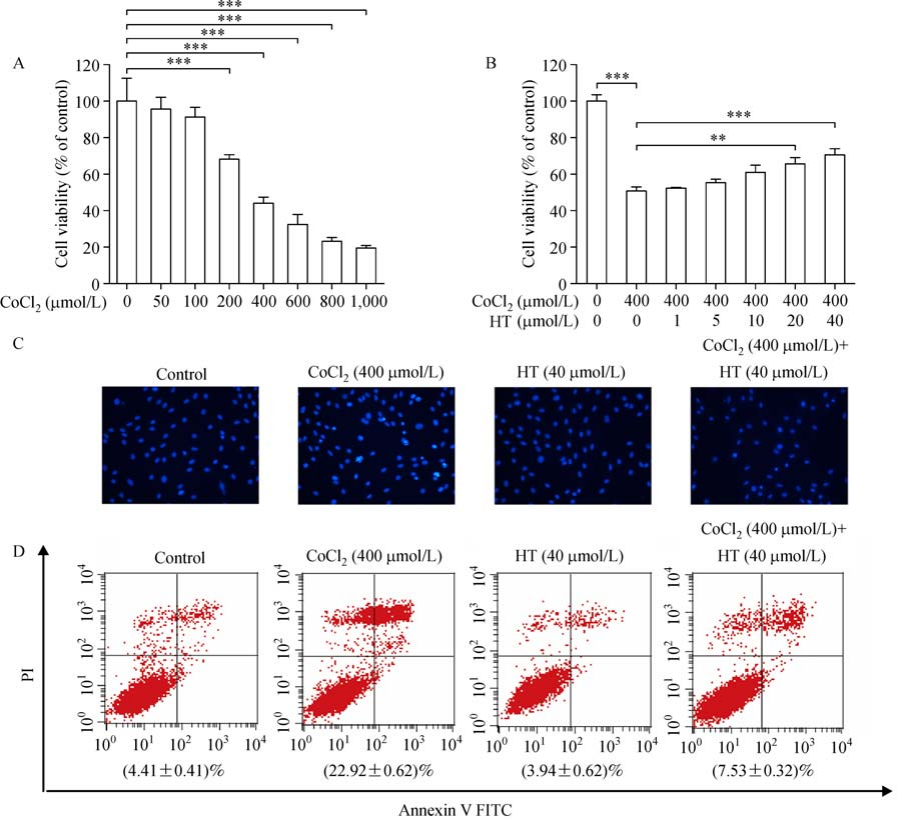
Hydroxytyrosol (HT) protects against CoCl_2_ induced apoptosis.

H9c2 cells were treated with CoCl_2 _at different concentrations (0–1,000 μmol/L) for 24 hours to induce hypoxia. As shown in ***Fig. 1A***, the cell viability revealed a dose-dependent decrease. When the concentration reached 200 μmol/L, CoCl_2_ significantly decreased cell viability. Because of the cell viability of 400 μmol/L CoCl_2_ treatment was about 50%, this concentration of CoCl_2_ was used to induce cell hypoxia in subsequent experiments. When the cells were pretreated with hydroxytyrosol (***Fig. 1B***) for 24 hours and then treated with CoCl_2_ for 24 hours, cell viability recovered in a concentration-dependent way. At 40 μmol/L hydroxytyrosol, significantly increased cell viability was observed, compared with CoCl_2_ treatment.

To confirm the results, Hochest 33258 staining was used to detect apoptotic cell death. H9c2 cells in the control group showed weak fluorescent spots, whereas the cells exposed to 400 μmol/L CoCl_2_ for 24 hours showed strong fluorescent spots; furthermore, pretreatment of hydroxytyrosol reversed the apoptosis of H9c2 cells induced by CoCl_2_. These indicate the protective role of HT against the damage effect of CoCl_2_ (***Fig. 1C***). In addition, to further confirm this effect, Annexin-V FITC/propidium iodide staining with flow cytometry was performed, which also showed that hydroxytyrosol could alleviate H9c2 apoptosis induced by CoCl_2_ (***Fig. 1D***).

### Expression of GRP78 and CHOP after CoCl_2_ treatment

**Fig.2:**
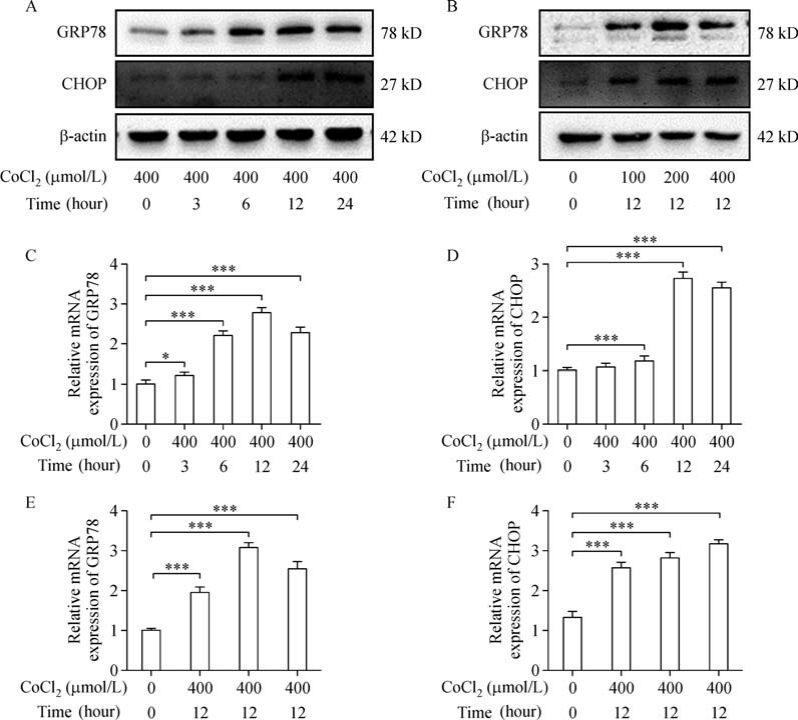
The expression of GRP78 and CHOP after CoCl_2 _ treatment.

The induction of hypoxic stress was in parallel with induction of GRP78 and CHOP^[19]^. Western blotting analysis showed that 400 μmol/L CoCl_2_ markedly increased the expression of GRP78 time-dependently at 0-12 hours. At 24 hours, the expression of GRP78 slightly decreased (***Fig. 2A***). The expression of CHOP showed a time-dependent increase at 0–24 hours. When the cells were treated with CoCl_2_ at various concentrations for 12 hours, the expression of GRP78 peaked at 200 µmol/L and then decreased at 400 µmol/L. Besides, the expression of CHOP was increased significantly in a dose-dependent manner (***Fig. 2B***). As expected, the expression of GRP78 and CHOP in mRNA were consistent with change of protein at different time points and concentrations (***Fig. 2C***, ***D***, ***E*** and ***F***).

### Hydroxytyrosol inhibited the expression of GRP78 and CHOP after CoCl_2_ treatment

**Fig.3:**
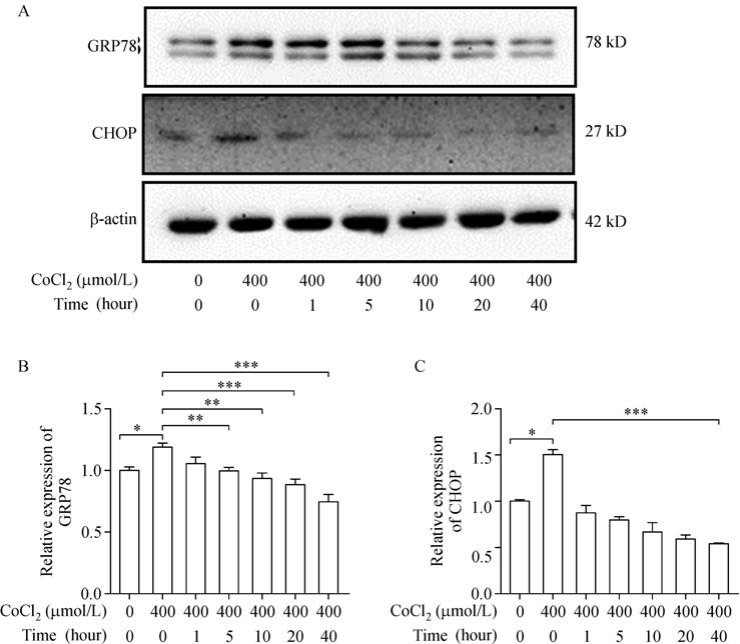
Hydroxytyrosol inhibits the expression of GRP78 and CHOP after CoCl_2 _ treatment.

According to the above results, HT could protect H9c2 cells against the damage of CoCl_2_. CoCl_2_ treatment could increase the expression of GRP78 and CHOP, which might be a result of hypoxic stress. As shown in ***Fig. 3A***, H9c2 cells were pretreated with hydroxytyrosol at the indicated concentrations for 24 hours, and then treated with CoCl_2_ (400 µmol/L) for 12 hours. The expression of GRP78 and CHOP was inhibited dramatically by hydroxytyrosol, as compared to the treatment of CoCl_2_. In order to confirm the results, the mRNA levels of GRP78 and CHOP were detected. ***Fig. 3B–C*** shows that the mRNA levels of GRP78 and CHOP were upregulated. However, hydroxytyrosol at 10–40 μmol/L could reverse GRP78 levels significantly, and CHOP levels at 1–40 μmol/L. Therefore, the protective effects of hydroxytyrosol against the damage of CoCl_2_ might result from the inhibition of GRP78 and CHOP expression.

### Protective effects of OLE against isoproterenol-induced MI in rats

**Fig.4:**
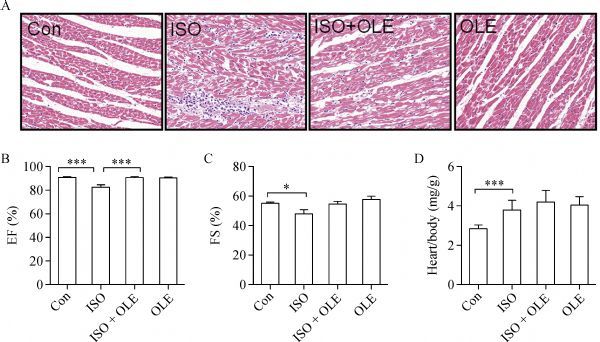
OLE exerts cardioprotection on rats subjected to isoproterenol .

According to the *in vitro* results, we evaluated the protective effects of OLE against isoproterenol-induced MI *in vivo*. OLE [200 mg/(kg·day)] was administered by gavage for one month before subcutaneous injection of isoproterenol [85 mg/(kg·day)] for two days. H&E staining displayed that rats receiving OLE treatment had normal cardiac fibers and no inflammatory cell infiltration. Rats receiving isoproterenol showed infarction areas, characterized by disordered cardiac muscle fibers and infiltration of inflammatory cells. However, after the pretreatment with OLE, these pathological changes were dramatically ameliorated (***Fig. 4A***).

As shown in ***Fig. 4B–C***, echocardiography showed that heart function in isoproterenol-treated rats was significantly decreased in ejection fraction and fraction shortening. However, OLE treatment considerably improved heart function with increased ejection fraction and fraction shortening. The heart weight over body weight ratio was higher in the isoproterenol group; however, this increase could be reversed by OLE (***Fig. 4D***).

### Protective effect of OLE on isoproterenol-induced MI through upregulating GRP78 and CHOP in rats

**Fig.5:**
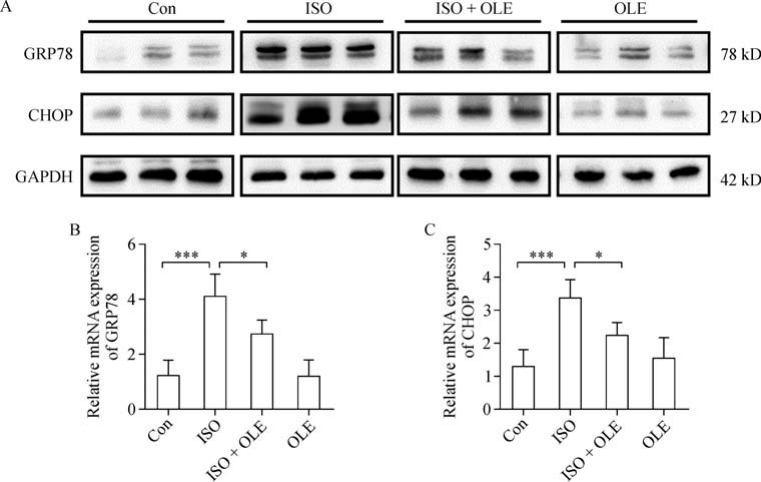
Effects of OLE on protein and mRNA levels of GRP78 and CHOP in rats.

Expression levels of GRP78 and CHOP *in vivo* were also detected. As expected, proteins extracted from hearts tissues treated with OLE [200 mg/(kg·day)] showed a low expression of GRP78 and CHOP, which was similar to that of the control group. However, isoproterenol [85 mg/(kg·day)] could enhance the expression of GRP78 and CHOP significantly. When treated with isoproterenol and OLE, the expression of these two proteins was reversed dramatically (***Fig. 5A***).

**Fig.6:**
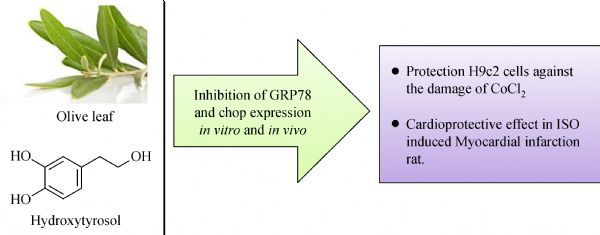
GRP78 and CHOP are involved in cardioprotective effect of hydroxytyrosol and OLE A schematic summary for the protection and mechanisms of hydroxytyrosol and olive leaf extract on myocardial infarction ***in vitro*** and ***in vivo***.

The mRNA levels of GRP78 and CHOP in heart tissues were also detected by quantitative RT-PCR. The expression levels of CHOP and GRP78 were higher in the group treated with isoproterenol [85 mg/(kg·day)] than that in control and OLE [200 mg/(kg·day)] treatment groups. However, combination of isoproterenol with OLE [200 mg/(kg·day)] could reverse the high expression of CHOP and GRP78 induced by isoproterenol [85 mg/(kg·day)] (***Fig. 5A ***and ***B***). Similar results were observed in the protein expression. These results demonstrated that the protection of OLE against isoproterenol-induced MI *in vivo* involved the inhibition of GRP78 and CHOP expression. As shown in ***Fig. 6A***, OLE and the main component, hydroxytyrosol can protect MI induced by isoproterenol in vivo and H9c2 cells hypoxia induced by CoCl2 *in vitro*.

## Discussion

This study elaborated that OLE can reverse isoproterenol-induced MI and its major component hydroxytyrosol can protect H9c2 cells against apoptosis induced by CoCl_2_ through the ER stress pathway *in vivo* and *in vitro*. To our knowledge, it is the first report that hydroxytyrosol attenuates CoCl_2_-induced hypoxia in H9c2 cells and OLE reverses MI through regulating molecules GRP78 and CHOP in the ER stress pathway.

CoCl_2_ is a well-known inducer of hypoxia in cultured cardiomyocytes. Also, isoproterenol is extensively used to induce acute MI in animal models that mimic myocardial ischemic-anoxic injury^[20–24]^. In our study, CoCl_2_ induces the apoptosis of H9c2 cells and reduces their viability in a dose-dependent manner, which is consistent with the other reports that CoCl_2_ induced hypoxia and apoptosis of H9c2 cells^[25–26]^. However, this is the first report of the antagonistic effect of hydroxytyrosol on CoCl_2 _showing hydroxytyrosol protected H9c2 cells against the cytotoxicity of CoCl_2_. Besides, xanthine/xanthine oxidase has been suggested as a risk factor for heart disease. Xanthine/xanthine oxidase induces cardiomyocyte apoptosis and death through generating superoxide and hydrogen peroxide^[27]^. In contrast, CoCl_2_ can replace ferrous ions of proline hydroxylase, which leads cells into a hypoxic state. Moreover, hydroxytyrosol maintains cardiomyocyte viability through inhibiting intracellular ROS generation and mediating the phosphorylation of survival or death related proteins in H9c2 cells subjected to xanthine/xanthine oxidase-induced oxidative stress^[27]^. *In vivo*, when the rats were treated with isoproterenol, MI was obviously induced as ejection fraction, fractional shortening, disarranged cardiac muscle fibers and inflammatory cell infiltration decreased, which is consistent with the finding of previous studies^[28–29]^.

The ER is a crucial component of the cellular reticular network that allows cells to adjust to a variety of conditions^[30]^, like protein folding, calcium homeostasis, and lipid biosynthesis. Stimuli such as oxidative stress and ischemic insults enhance the expression of normal and/or folding-defective proteins, leading to the accumulation of unfolded proteins. This phenomenon is called ER stress that activates unfolded protein reaction^[31]^. Three major branches of ER stress sensors are protein kinase ER-like kinase (PERK), inositol-requiring protein 1 (IRE1) and activating transcription factor 6 (ATF6), which are involved in unfolded protein reaction^[32]^. Under the normal condition, the ER stress sensors are bound to GRP78 and inactivated. But when stimulated, GRP78 is dissociated from ER sensors, ER sensors are activated, and unfolded protein reaction is initiated to reduce cellular dysfunction and promote cell survival. However, excessive unfolded protein reaction will initiate CHOP-mediating cell apoptosis^[33]^. Studies show that ER stress plays an important role in the pathogenesis of MI, ischemic heart diseases and heart failure^[30, 34–35]^. Suppressing ER stress and associated apoptosis provides protective effects on cardiovascular system^[36]^. In addition, ER stress induces the expression of CHOP and then inhibits the expression of anti-apoptotic proteins Bcl-2 and Bnip3^[37–38]^. In our study, we observed CoCl_2_ could induce the overexpression of CHOP and GRP78 *in vitro* and isoproterenol induced the similar results *in vivo*. Pretreated with hydroxytyrosol or OLE, the abnormal expression of CHOP and GRP78 was inhibited significantly.

The therapeutic effect of OLE and its main constituent hydroxytyrosol is notable. The formation of infarction, disordered cardiac muscle fibres and infiltration of inflammatory cells are the typical pathological characteristics of MI^[39]^. All of these pathologic changes could be ameliorated by OLE significantly.

In conclusion, this study demonstrates that OLE and its main constituent hydroxytyrosol can prevent H9c2 cell damage and MI induced by CoCl_2_ and isoproterenol. Defense ER stress is involved in this cardioprotective mechanism. The expression of GRP78 and CHOP, specific markers of ER stress, can be decreased significantly by OLE and hydroxytyrosol. Therefore, the OLE and hydroxytyrosol are effective and promising agents for treatment of MI. However, detailed molecular mechanisms of OLE and hydroxytyrosol still need to be studied in order to confirm these protective functions.
